# Dietary behaviors of rural residents in northeastern China: implications for designing intervention information and targeting high-risk population

**DOI:** 10.3389/fpubh.2024.1239449

**Published:** 2024-01-23

**Authors:** Li Bai, Haiheng Tang, Mingliang Wang

**Affiliations:** ^1^School of Biological and Agricultural Engineering, Jilin University, Changchun, China; ^2^Key Laboratory of Bionic Engineering, Ministry of Education, Jilin University, Changchun, China; ^3^School of Business and Management, Jilin University, Changchun, China

**Keywords:** dietary behavior, health literacy, nutrition knowledge, behavior intervention, health promotion

## Abstract

**Background:**

Dietary behavior is a pivotal modifiable determinant in reducing the occurrence of obesity/overweight and chronic non-communicable diseases. Improving the dietary behavior of rural residents in China is imminent due to the poor performance of their dietary behavior. Nutrition knowledge and health literacy are considered as elements that are linked intimately to healthy dietary behaviors but lack research in the Chinese setting.

**Purpose:**

The study is designed to explore the relationship between nutritional knowledge, health literacy and dietary behaviors and to analyze the performance under different demographic characteristics.

**Methods:**

A face-to-face survey of 400 rural residents on their nutrition knowledge, functional health literacy and dietary intake of five food categories consisting of 32 items was conducted based on a validated questionnaire. Descriptive analysis, difference test including ANOVA, *t*-test and non-parametric test, and multivariate linear regression were used for data analysis.

**Results:**

The results indicate that declarative nutrition knowledge, individuals’ information application capacity, and dietary behaviors, especially the intake of fruits, dairy and beans, and vegetable are not ideal and requires improvement. Male, elder, low-income, unmarried, and low-education populations performed significantly worse and were the high-risk group. Procedural nutrition knowledge, information access capacity, information understanding capacity, and information application capacity have remarkable effects on better dietary behavior.

**Conclusion:**

This study provides evidence-based guidance for prioritizing information and populations for healthy dietary interventions.

## 1 Introduction

The Chinese population is switching from a plant-based diet to western-style diets high in fat and animal-based foods (1), and malnutrition has significantly decreased. But, the proportion of residents with an imbalanced diet and overnutrition has gradually increased, and there is a significant expansion in the prevalence of overweight, obesity and patients with chronic non-communicable diseases (2). The proportion of adults with a BMI ≥ 25 kg/m^2^ have increased from 20% (about 150 million) in 1992 to nearly 50% (about 550 million) in the most recent national survey (3). Dietary behavior is one of the key modifiable determinants for reducing obesity/overweight and chronic disease prevalence in China (4, 5). The Chinese government pays significant attention to improving the residents’ dietary and successively formulated the Outline of the Health China 2030 Plan (6) and the National Nutrition Plan (2017–2030) (7).

Currently, the incidence of chronic diseases is higher in urban areas than in rural areas in China, and the growth rate of rural areas is higher than that in urban areas, which is related to poor dietary behavior in rural residents (8). China, however, is a developing country with a rural population of over 500 million, and policy implementation capacity in rural areas is extremely limited (9). It is necessary to identify priority intervention contents and groups in order to conduct interventions more effectively.

To prioritize the intervention information, two domains that are closely associated to dietary behavior, nutritional knowledge and health literacy, were considered. The prevailing research state that nutrition knowledge has an important role in health behavior (10, 11), including dietary behavior (12, 13). But some intervention experiments based on knowledge courses have yielded mixed results, and such differences may be due to interventions targeting different types of knowledge. Some studies have indicated that knowledge is not a simple structure, but multiple types (14). In cognitive psychology, knowledge can be classified as declarative knowledge or procedural knowledge. Declarative knowledge is knowing about facts and objects, while procedural knowledge is pertaining to the execution of behavior (15). Several scholars have analyzed the relationship with dietary behavior using procedural nutrition knowledge (PNK) and declarative nutrition knowledge (DNK), respectively (16, 17), but limited studies have considered both types of knowledge, especially for Chinese populations.

Health literacy has become increasingly important in recent years because evidence supports its association with healthy behaviors (18). Health literacy also plays an important role in health education and promotion (19) and is negatively correlated with national healthcare utilization and expenditure (20). The U.S. Department of Health and Human Services has defined health literacy as the ability of individuals to access, process, and understand health information to make decisions about treatment and their health in general (21). Regarding dietary behaviors, some studies have found that health literacy motivates individuals to make healthier dietary food choices (22, 23), but others have demonstrated the reverse effect (18). The role of health literacy in the promotion of dietary behavior of the Chinese residents remains to be clarified. Functional health literacy (FHL) is the most common type of health literacy assessment, and this study is based on the FHL scale to evaluate individuals’ capacity to access, understand, and apply health information (24–26).

Priority intervention populations were identified by examining differences in nutrition knowledge, health literacy, and dietary behavior between groups. Previous studies have indicated that FHL, nutrition knowledge, and dietary behavior are affected by demographic characteristics. Characteristics such as gender (27), age (28), region (urban or rural) (29), occupation (30), education level, household income, and self-reported health status (31) were found to affect health literacy. Xu et al. (32) found that significant differences in nutritional knowledge across demographic characteristics, which in turn influenced their dietary habits and health. Education level, gender, BMI, and exposure to chronic disease were found to influence individuals’ nutritional knowledge and dietary behavior (32–35). To identify priority intervention populations in a multi-dimensional way, a wide range of demographic variables, including gender, age, education, marital status, income, body mass index (BMI), and chronic disease status, were selected to identify high-risk groups.

**Figure 1:**
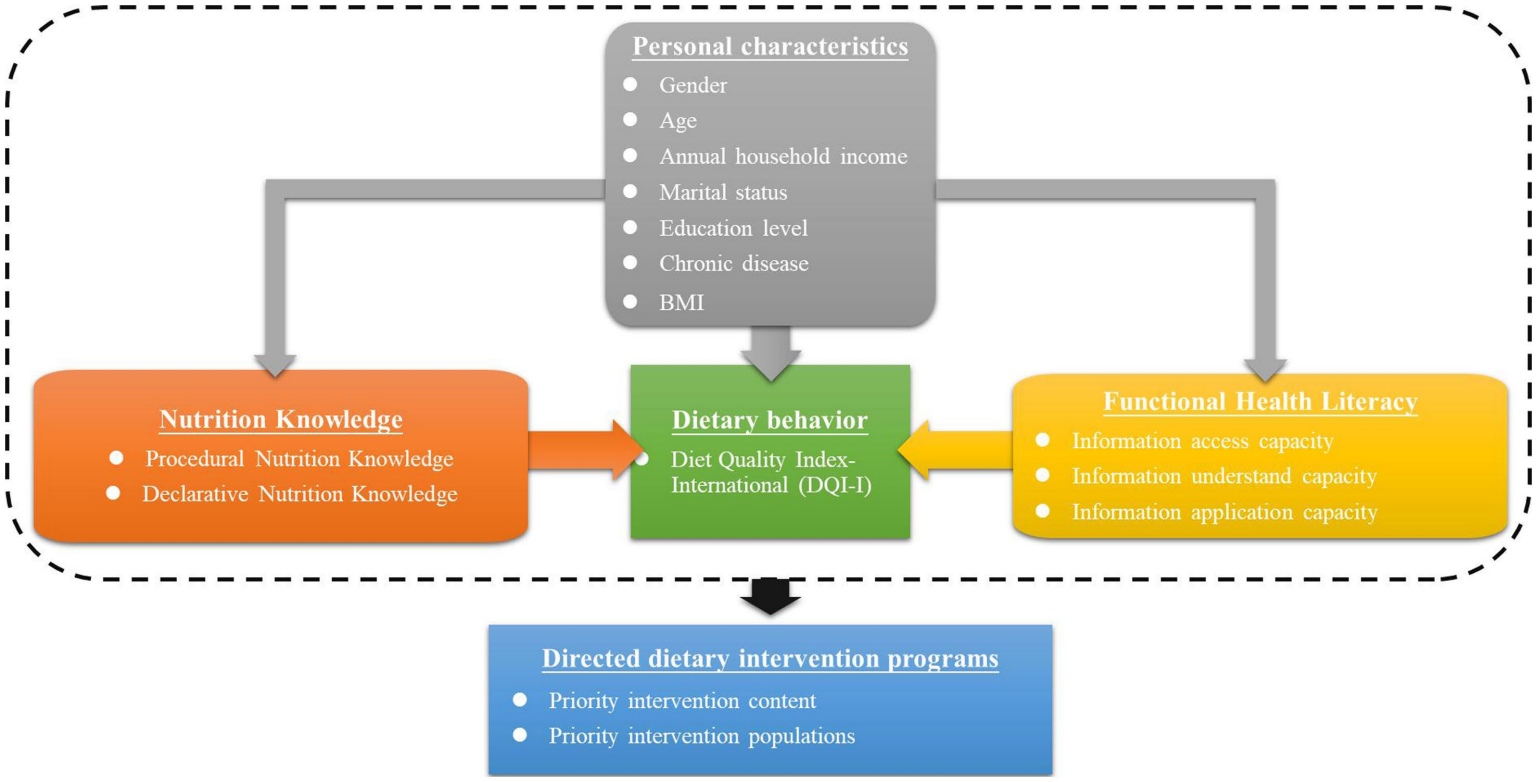
Research framework.

In summary, this study collected data on the nutrition knowledge, FHL, and dietary behavior of rural residents in China to serve three objectives: (1) to assess the current dietary behavior, FHL, and nutrition knowledge of rural residents in China; (2) to examine the differences in dietary behavior, FHL, and nutrition knowledge between different populations; and (3) to analyze the relationship between health literacy and nutrition knowledge and dietary behavior. This study provides guidelines for determining the priority intervention information and populations for healthy dietary intervention programs in rural China. Figure 1 shows the research framework.

## 2 Materials and methods

### 2.1 Questionnaire design

To investigate the nutrition knowledge, FHL, and dietary behavior, we designed a questionnaire for the target population. The questionnaire in this study had four parts: (1) demographic characteristics; (2) nutrition knowledge; (3) FHL; and (4) a food frequency questionnaire (FFQ). Demographic characteristics included gender, age, marital status, education level, annual household income, BMI, and chronic disease status. Nutrition knowledge included the PNK and DNK, and their items are from the studies of Dickson-Spillmann et al. (36) and Ju (37), respectively. The form of categorical judgment was chosen as the response to the nutrition knowledge, and all nutrition knowledge questions had only one correct answer. FHL included information access capacity (IACC), information understanding capacity (IUC), and information application capacity (IAPC). We selected the FHL questions from the health literacy questionnaire proposed by Osborne et al. (38) and FHL was measured using a five-point Likert scale. The original PNK and FHL questions were written in English. The questions were translated into Chinese, and two bilingual experts on dietary behavior corrected the semantics of the questionnaire and ensured translation equivalence. An initial set of items was formed consisting of seven items for measuring PNK, six items for measuring DNK, and nine items for measuring FHL.

The FFQ was used as the *dietary intake questionnaire* to investigate food consumption frequency and intake in the past week in the subject. Food was divided into five categories (i.e., meat, poultry, fish, and eggs; dairy and beans; grain; fruit; and vegetable), and specific food names were listed in every category as a reference for the subject to fill in the food consumption frequency and food intake. To increase the suitability of the questionnaire, the types of food consumed with high frequency in the survey area were pre-survey by a group of 30 rural residents. The 24 most commonly consumed foods were listed in the food frequency questionnaire, and a blank area was present for the subject to add in foods that were not mentioned. Two forms were used for food consumption frequency. Foods with a high consumption frequency were written as “X times/day,” and foods with a low consumption frequency were written as “W times/week (7 days).” The question was not filled in if the food was not consumed in the past week. When filling in the food intake, the *schematic diagram of standard food weight* provided by the investigator was used to estimate the weight of the food consumed each time. See Appendix for *schematic diagram of standard food weight* (Supplementary Figure S1) and *dietary intake questionnaire* (Supplementary Table S1).

Three instruments, i.e., content validity, internal consistency, and test–retest reliability, were applied to evaluate the reliability of the questionnaire. First, an expert group was organized to assess the content validity of the questionnaire. The group is composed of two professors, one assistant professor, and three doctoral candidates, whose research areas are focused on nutrition and consumer behavior. Explain to the expert group the study objectives, questionnaire content, and assessment procedure via email or WeChat (a kind of instant messaging social software), and invite participation after receiving consent (39). The assessment included question clarity and understanding, relationship to the study objective, overlap between questions, answer rationality, and conformance to China’s context. In the light of the expert group’s comments, we deleted some questions that were not clearly stated. For example, one item which asked *whether it was unhealthy to eat an excessive proportion of fruits and vegetables in the daily diet* was deleted because it mentioned the intake of both fruits and vegetables, which is a double-barrelled question, and this may have confused respondents. Some questions were optimized, i.e., the item “*A balanced diet is one that has the same intake of all nutrients*” was modified to “*A balanced diet is one that has the same intake of all types of food*,” as nutrients are academic jargon that are difficult to understand for rural residents. Subsequently, a preliminary study was carried out on 30 rural residents. Each respondent completed the questionnaire from 30 to 45 min. We used Cronbach’s α coefficient to assess the internal consistency. Cronbach’s α coefficient of FHL (including information access capacity, information understanding capacity, and information application capacity) was above 0.8, and the Cronbach’s α coefficients of PNK and DNK were 0.597 and 0.538, respectively. In some studies involving the internal consistency of the items, especially for knowledge items, it was considered acceptable if it was close to the threshold (α = 0.6) (40, 41). Nunnally (42) first version of the introduction to Cronbach’s α coefficients suggested that the minimum accepted ranges in preliminary studies were 0.5 to 0.6. Thus, the internal consistency of items measuring the FHL, PNK, and DNK is acceptable. To further evaluate the stability of the questionnaire over time, 20 subjects were randomly selected for a telephone interview 3 days after the first investigation, and 10 questions in the questionnaire were randomly selected for the interviewee to answer. The two investigations were conducted 3 days apart to decrease memory bias, and 10 questions were randomly selected to reduce sequence bias. The Pearson correlation coefficient of the two scores was 0.86 (*p* < 0.01), showing that the questionnaire has high test–retest reliability.

### 2.2 Data collection

#### 2.2.1 Survey site

Three provinces in northeastern China, which are Heilongjiang, Jilin, and Liaoning, were selected as survey areas. Northeastern China has the highest latitude and cold climate, and these three provinces are the main grain-producing regions of China and are major agricultural provinces, which have shaped unique dietary patterns. The daily diet of residents in northeastern China contained excess salt, and long-term high salt intake is an important factor causing chronic diseases (43, 44). Moreover, due to the long and cold winters in northeastern China, residents usually consume grains and meat with high calorific value to withstand the cold weather, leading to a lower proportion of fresh fruits and vegetables in their diet. Hu et al. (45) found that increasing fruit and vegetable intake in northeastern China could decrease the risk of lung cancer. Considering the high health risks associated with this unique dietary pattern, there is a great tendency to conduct healthy eating interventions in these regions.

#### 2.2.2 Questionnaire survey

Ten investigators were recruited to conduct the survey. The survey was carried out in the form of face-to-face interviews. To ensure the credibility and reliability of the survey, training for investigators was implemented. The training included an introduction to the purpose of the survey, an explanation of the meaning of each question and how it should be answered, procedures for conducting the survey, and tips for dealing with unexpected situations that they may encounter during face-to-face interviews. The survey was conducted in the homes of the respondents. To ensure the representativeness of the sample, ten villages in three provinces were selected as survey locations by stratified random sampling. The sampling pattern is to select one city in each province and the corresponding village in each city. As Liaoning Province has the largest number of residents living in rural areas, four villages were selected in Liaoning Province and three villages were selected in the other two provinces. Forty residents in each village were recruited to conduct the survey. To increase randomness, one family was chosen to visit in every geographically separated 3 households. The questionnaire survey was completed from June to August 2019.

Based on the Bartlett et al. (46) formula shown below, the required sample size was estimated to be 323. Given the non-response rate, a total of 400 questionnaires were distributed (Eq. 1).


(1) $$n=\frac{\frac{Z_{\frac{\alpha }{2}}^{2}pq}{d^{2}}}{1+\frac{1}{N}\left(\frac{Z_{\frac{\alpha }{2}}^{2}pq}{d^{2}}-1\right)}$$

where n denotes the required sample size, d denotes a 5% margin with error (the standard value = 0.05), *Z* denotes a 95% confidence interval $\left(Z_{\frac{\alpha }{2}}=1.96\right)$, *p* denotes the proportion of the target population (*p* = 0.7), and *q* denotes *1 − p* (*q* = 0.3). *N* represents the population size, which, according to the 2018 statistics, is 34.84 million rural residents in the three provinces of Northeast China, i.e., *N* = 348,400,000.

Three hundred and seventy questionnaires were returned, of which 344 were valid questionnaires with no missing values, with a response rate of 92.5% (370/400) and a validity rate of 93.0% (344/370). Three hundred and forty-four valid samples are above the threshold number of required sample size (*n* = 323).

### 2.3 Data analysis

Amos (version 22.0) and SPSS (version 23.0) were applied for statistical analysis. The frequency (count and percentage) of every option was calculated, and the mean score and standard deviation (S.D.) of every question was calculated. Kurtosis and skewness were used to test the normality of the variables. It is considered that the variables are normally distributed when the absolute values of kurtosis index and skewness index are less than 7 and 2, respectively (47). ANOVA and *t*-test was used to compare the differences when data were normally distributed and variance was homogeneous. In other situations, nonparametric tests (Mann–Whitney U test or Kruskal–Wallis test) were used. Ordinary least squares (OLS) were used to analyze the relationship between nutrition knowledge, FHL, and dietary behavior. Variance inflation factor (VIF) was applied to check the multicollinearity of each fitted model (48).

For knowledge questions, 1 point and 0 point were given for a correct and incorrect answer, respectively. The PNK score was from 0 to 7 and the DNK score was from 0 to 6, and total nutrition knowledge was from 0 to 13. The five-point Likert scale was scored ranging from one to five. The FHL scale contains 9 items with scores ranging from 9 to 45. The three subscales, IACC, IUC, and IAPC, each contained three items scored from 3 to 15. Referring to Nakayama et al. (49), four levels of health literacy were defined based on average FHL scores: 9–23 as inadequate, 24–31 as problematic, 32–38 as sufficient, and 39–45 as excellent.

The scientific community has given diverse instruments for diet assessment from different perspectives. The Dietary Quality Index-International (DQI-I), one of the composite measures that enable a more precise identification of the relationship between overall diet quality scores and the risk of diet-related diseases, was adopted because it is considered a valid tool to make comparisons across countries and regions (50–52). Notably, the DQI-I in the study was calculated depending on the intake of the five food groups in accordance with the recommended values without reference to micronutrients and macro-nutrients. The reason for this calculation format is that the dietary pattern recommended by the Chinese Dietary Guidelines mainly focuses on the intake of these five food groups, and that the adequate intake of these five food groups may, to a certain extent, meet the body’s needs for various types of nutrients and reduce chronic diseases (53). The first category (meat, poultry, fish, and eggs) included eight types of foods; the second category (dairy and beans) included five types of foods; the third category (grain) included seven types of foods; the fourth category (fruit) included six types of foods; and the fifth category (vegetable) included six types of foods. The daily intake for each food category ($y_{i}$ ) was equal to the sum of all food intake under that category. Refer to Eqs. (2–6). For high consumption frequency food, the respondent would write the number of times the food was consumed each day ($X_{i}$ ) and the amount of each intake ($M_{i}$ ). For less consumption frequency food, the respondent would write the number of times the food was consumed each week ($W_{i}$ ) and the amount of each intake ($M_{i}$ ), *y_i_* is the daily intake of each category of food:


(2) $$y_{meat,poultry,fish,andeggs}=\overset{i=8}{\underset{i=1}{\sum }}\left(M_{i}\times X_{i}+M_{i}\times W_{i}/7\right)$$

(3) $$y_{dairyandbeans}=\overset{i=13}{\underset{i=9}{\sum }}\left(M_{i}\times X_{i}+M_{i}\times W_{i}/7\right)$$

(4) $$y_{grain}=\overset{i=20}{\underset{i=14}{\sum }}\left(M_{i}\times X_{i}+M_{i}\times W_{i}/7\right)$$

(5) $$y_{fruit}=\overset{i=26}{\underset{i=21}{\sum }}\left(M_{i}\times X_{i}+M_{i}\times W_{i}/7\right)$$

(6) $$y_{vegetable}=\overset{i=32}{\underset{i=27}{\sum }}\left(M_{i}\times X_{i}+M_{i}\times W_{i}/7\right)$$

The 2022 Chinese Dietary Guideline recommends that the recommended intake for five food categories (i.e., meat, poultry, fish, and eggs; dairy and beans; grain; fruit; and vegetable) is 150 g/day, 300 g/day, 250 g/day, 200 g/day, and 300 g/day, respectively. If the intake of a category was lower than the recommended intake; it was scored as 0; otherwise, it was scored as 3. The range of DQI-I is from 0 to 15.

## 3 Results

### 3.1 Profile of samples

The sample characteristics of the survey is shown in Table 1. There were slightly more female respondents (57%) in the sample than male respondents (43%), 59% of respondents were between the ages of 35 and 54 years old, nearly 50% of the respondents having an annual household income range from RMB 36,000–84,000, equivalent to US$ 4,956–11,565.^1^ The proportion of married respondents (73%) in the sample is substantial, and more than 50% of the respondents have a junior high school education and below, which is consistent with the lack of young and well-educated people in rural China (54). A significant proportion of the sample, about 36%, were suffering from chronic diseases, and more than 50% of the respondents were not at a healthy weight, which is consistent with the results of previous studies that suggest that the health status of rural residents in Northeast China is a matter of concern (55).

**Table 1:** Participant characteristics (*n* = 344).

Characteristics		*n*	%
Gender	Male	148	43
	Female	196	57
Age	18–34	97	28.2
	35–44	108	31.4
	45–54	95	27.6
	Above 55	44	12.8
Annual household income	Below 36,000	129	37.5
	36,000–84,000	170	49.4
	Above 84,000	45	13.1
Marital status	Unmarried	79	23
	Married	251	73
	Other	14	4.1
Education	Primary and below	72	20.9
	Junior high school	111	32.3
	High school	94	27.3
	Junior college or above	67	19.5
Chronic disease	Yes	125	36.3
	No	219	63.7
BMI	Underweight (< 18.5)	50	14.5
	Healthy weight (18.5–23.9)	171	49.7
	Overweight (24 to 27.9)	78	22.7
	Obesity (≥28)	45	13.1

### 3.2 Descriptive data analysis results

As shown in Table 2, the mean PNK score was 4.66 (range from 0 to 7), and the question with the lowest accuracy rate was *meat should be the basis of our daily diet* (45.9%), which the highest accuracy rate was *fat is always bad for your health, so you should avoid it as much as possible* (70.6%). The mean DNK score was 2.53 points (range from 0 to 6), and the question with the lowest accuracy rate was *daily salt intake should not exceed* (27.6%), which the question with the highest accuracy rate was *which of the following food groups contains the most protein* (65.7%). The mean score of total nutritional knowledge was 7.19 (range from 1 to 13).

**Table 2:** Nutrition knowledge passing rate and mean score (*n* = 344).

Items	Options	*n*	%
*Procedural nutrition knowledge (Min = 0 Max = 7 Mean = 4.66 S.D. = 1.74 Skew. = − 0.538 Kurt. = − 0.553)*			
1. Fruit can be fully replaced by vitamin and mineral supplements	A. Agree	54	15.7
	**B. Disagree**	**228**	**66.3**
	C. Not sure	62	18.0
2. A healthy diet means nothing other than eating vitamins	A. Agree	61	17.7
	**B. Disagree**	**235**	**68.3**
	C. Not sure	48	14.0
3. Meat should be the basis of our daily diet	A. Agree	145	42.2
	**B. Disagree**	**158**	**45.9**
	C. Not sure	41	11.9
4. Instead of eating fruit you can drink fruit juice	A. Agree	68	19.8
	**B. Disagree**	**238**	**69.2**
	C. Not sure	38	11.0
5. Fat is always bad for your health, so you should avoid it as much as possible	A. Agree	44	12.8
	**B. Disagree**	**243**	**70.6**
	C. Not sure	57	16.6
6. A balanced diet implies eating all foods in the same amounts	A. Agree	55	16.0
	**B. Disagree**	**228**	**66.3**
	C. Not sure	61	17.7
7. For healthy nutrition, dairy products should be consumed in the same amounts as fruit and vegetables	A. Agree	75	21.3
	**B. Disagree**	**177**	**51.5**
	C. Not sure	92	26.7
*Declarative nutrition knowledge (Min = 0 Max = 6 Mean = 2.53 S.D. = 1.51 Skew. = 0.237 Kurt. = − 0.638)*			
1. The units of heat, KCAL (kilocalorie) and KJ (kilojoule) are the same	A. Agree	38	11
	**B. Disagree**	**130**	**37.8**
	C. Not sure	176	51.2
2. The reasonable supply of three meals a day requires that the proportion of calories for breakfast, lunch and dinner is	A. 20%, 50%, 30%	49	14.2
	**B. 30%, 40%, 30%**	**154**	**44.8**
	C. 30%, 30%, 40%	40	11.6
	D. Not sure	101	29.4
3. The main nutrient provided by animal food is	**A. Protein**	**140**	**40.7**
	B. Carbohydrate	23	6.7
	C. Fibrin	118	34.3
	D. Vitamin	8	2.3
	E. Not sure	55	16
4. Which of the following foods is a major source of iron?	A. Milk	12	3.5
	B. Spinach	148	43
	**C. Animal liver**	**125**	**36.3**
	D. Orange	10	2.9
	E. Not sure	49	14.2
5. Daily salt intake should not exceed	A. 3 g	49	14.2
	B. 5 g	72	20.9
	**C. 6 g**	**95**	**27.6**
	D. 8 g	26	7.6
	E. Not sure	102	29.7
6. Which of the following food groups contains the most protein	A. Red beans, milk, mung beans	32	9.3
	**B. Milk, chicken, fish**	**226**	**65.7**
	C. Meat, lettuce, beans	35	10.2
	D. Bread, beef, spinach	10	2.9
	E. Not sure	41	11.9
Total nutrition knowledge (Min = 0 Max = 13 Mean = 7.19 S.D. = 2.59 Skew. = − 0.085 Kurt. = − 0.396)			

Correct answers are shown in bold.

**Figure 2:**
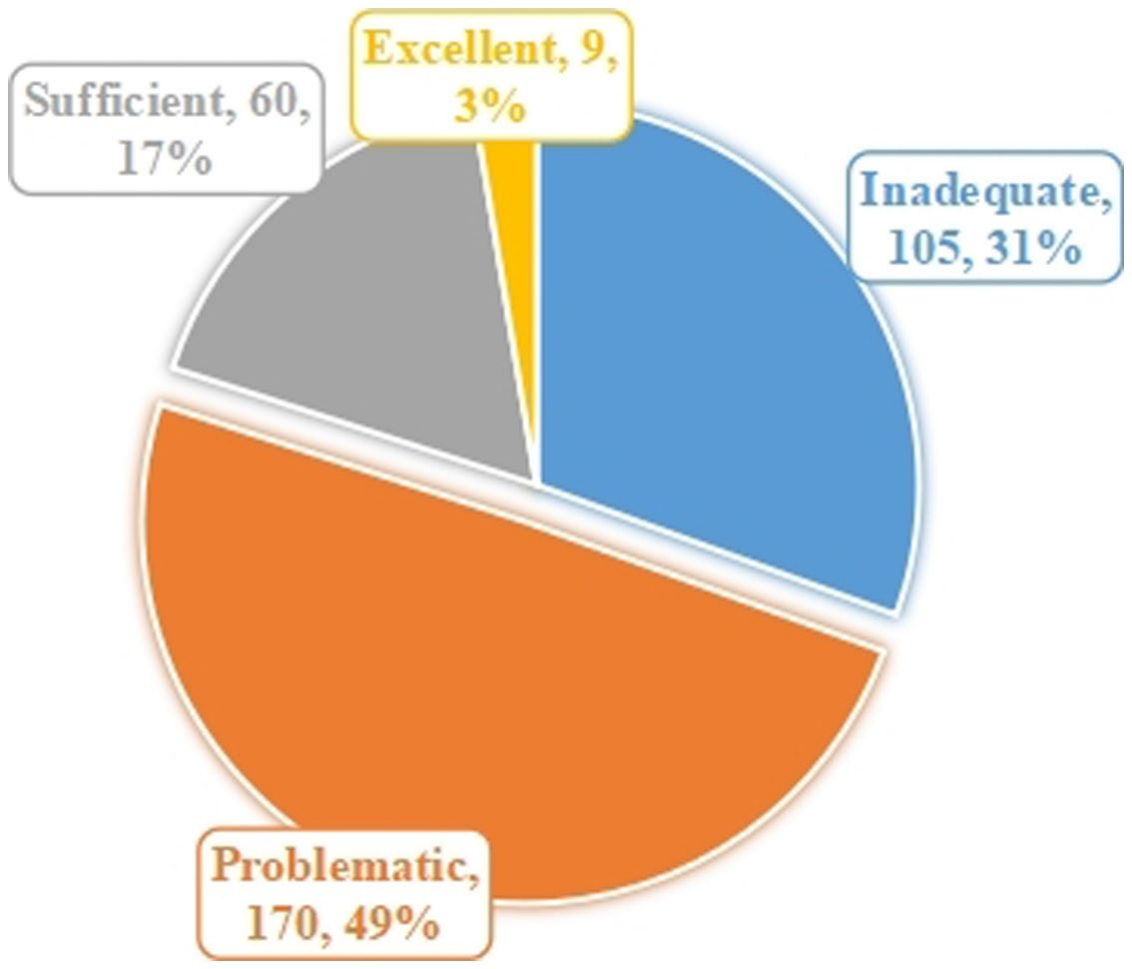
Scores on functional health literacy.

As shown in Table 3 and Figure 2, the average FHL score was 26.52 (range from 9 to 45, S.D. = 6.49), with 80% of respondents (*n* = 275) having a health literacy status of inadequate/problematic, and only 3% of respondents (*n* = 9) having excellent health literacy. For the three sub-structures of the FHL, the highest mean score was IACC (9.78 ± 3.55), followed by the IUC (9.19 ± 3.05), with the lowest mean score for the IAPC (7.55 ± 2.81). The question with the highest mean score was *I can obtain the healthy diet information that I need from text search* (3.35 ± 1.27), and the question with the lowest mean score was *I have sufficient healthy diet information to manage my diet* (2.49 ± 1.13).

**Table 3:** Descriptive statistics on functional health literacy (*n* = 344).

	Items	Min.	Max.	Mean	S.D.	Skew.	Kurt.
IACC^A^	IACC1 I have the ability to get information about healthy diet from different sources	1	5	3.15	1.28	−0.084	−0.984
	IACC2 I was able to find information about healthy diet that I was interested in	1	5	3.27	1.28	−0.289	−0.990
	IACC3 I can find healthy diet information by text	1	5	3.35	1.27	−0.374	−0.891
	Total	3	15	9.78	3.55	−0.227	−0.875
IUC^B^	IUC1 I can read and understand all the information about healthy diet	1	5	3.21	1.17	−0.316	−0.731
	IUC2 I was able to fully understand the information I was getting about healthy diet	1	5	2.89	1.22	0.081	−0.885
	IUC3 When I see information about healthy diet, I judge	1	5	3.09	1.17	−0.203	−0.812
	Total	3	15	9.19	3.05	−0.141	−0.672
IAPC^C^	IAPC1 I feel better informed about healthy diet	1	5	2.51	0.98	0.149	−0.543
	IAPC2 I have mastered enough healthy dietary information to manage my own diet	1	5	2.49	1.13	0.522	−0.458
	IAPC3 I have enough healthy diet information to help me achieve a healthy diet	1	5	2.55	1.14	0.435	−0.575
	Total	3	14	7.55	2.861	0.334	−0.662
FHL		10	44	26.52	6.491	−0.211	0.104

IACC, information access capacity; IUC, information understanding capacity; IAPC, information application capacity; FHL, functional health literacy. Different letters indicate significant differences in scores based on paired sample *t*-tests.

As shown in Table 4, the mean DQI-I score was 10.37 (range from 0 to 15). In particular, the proportion of respondents whose average daily intake of food categories met the recommended values at least four or more 50.87%. The percent of respondents whose average daily grain intake meet the recommendations was the highest, at 87.8%. While, only 55% of respondents ate the recommended amount of fruits and 62.8 and 67.7% of respondents ate the recommended amount of dairy and beans, and vegetable, respectively.

**Table 4:** Descriptive statistics of dietary behavior (*n* = 344).

Category	Meet the recommended intake	Not meet the recommended intake
Meat, poultry, fish, and eggs≥150 g	287 (83.4%)	57 (16.6%)
Dairy and beans≥300 g	216 (62.8%)	128 (37.2%)
Grain≥250 g	302 (87.8%)	42 (12.2%)
Fruit≥200 g	190 (55.2%)	154 (44.8%)
Vegetable≥300 g	233 (67.7%)	111 (32.3%)
	**Score**	**N (%)**
DQI-I Mean = 10.73 S.D. = 2.582 Skew. = − 0.001 Kurt. = −0.398	15	53 (15.4%)
	12	122 (35.5%)
	9	141 (41.0%)
	6	26 (7.6%)
	3	2 (0.6%)
	0	0 (0)

### 3.3 Difference analysis under different demographic variables

As shown in Table 5, the difference analysis of dietary knowledge of rural residents under different populations revealed that the overall nutrition knowledge level differed significantly by gender, annual household income, and education level. The PNK level of the residents varies as a function of the annual household income and education level. Three demographic dimensions, age, annual household income and education level, are important indicators for determining the different DNK levels of the residents.

**Table 5:** Relationship between demographic characteristics and nutrition knowledge, functional health literacy, and dietary behavior.

Characteristics		*N*	PNK	DNK	Nutrition knowledge	IACC	IUC	IAPC	FHL	DQI-I
Gender	Male	148	4.47 ± 1.77	2.38 ± 1.46	**6.85 ± 2.6**	10.05 ± 3.57	**8.74 ± 2.93**	**7.04 ± 2.8**	25.84 ± 6.62	10.52 ± 2.60
	Female	196	4.80 ± 1.57	2.64 ± 1.54	**7.44 ± 2.57**	9.57 ± 3.53	**9.53 ± 3.1**	**7.93 ± 2.85**	27.02 ± 6.51	10.88 ± 2.56
	*p*-value^A^		0.089	0.108	**0.037**	0.207	**0.018**	**0.004**	0.099	0.198
Age	18–34	97	4.91 ± 1.59	**2.78 ± 1.33a**	7.69 ± 2.36	9.64 ± 3.72	9.4 ± 3.14	7.28 ± 2.78	26.32 ± 7.4	10.76 ± 2.7
	35–44	108	4.57 ± 1.73	**2.51 ± 1.60ab**	7.08 ± 2.69	9.89 ± 3.58	9.16 ± 3.21	7.44 ± 2.95	26.49 ± 6.54	10.64 ± 2.73
	45–54	95	4.56 ± 1.90	**2.53 ± 1.54b**	7.08 ± 2.66	9.81 ± 3.47	9.34 ± 2.82	7.82 ± 2.95	26.97 ± 5.86	10.83 ± 2.33
	55 and above	44	4.52 ± 1.75	**2.02 ± 1.52b**	6.55 ± 2.61	9.73 ± 3.34	8.48 ± 2.93	7.8 ± 2.63	26 ± 6.38	10.64 ± 2.54
	*p*-value^C^		0.423	**0.038**	0.83	0.966	0.377	0.533	0.849	0.95
Annual household income	36,000 and bellow	129	**4.55 ± 1.84a**	**2.44 ± 1.56a**	**6.99 ± 2.77a**	9.58 ± 3.61	**8.46 ± 3.24a**	7.26 ± 2.98	**25.3 ± 6.91a**	10.4 ± 2.43
	36,000–84,000	170	**4.58 ± 1.72a**	**2.37 ± 1.32a**	**6.95 ± 2.3a**	9.79 ± 3.43	**9.66 ± 2.86b**	7.75 ± 2.81	**27.21 ± 6.2b**	10.99 ± 2.48
	84,000 and above	45	**5.24 ± 2.24b**	**3.38 ± 1.76b**	**8.62 ± 2.7b**	10.27 ± 3.8	**9.49 ± 2.86b**	7.58 ± 2.69	**27.33 ± 6.64b**	10.67 ± 3.23
	*p*-value^A^		**0.05**	**0.001**	**0.001**	0.536	**0.002**	0.341	**0.03**	0.137
Marital status	Unmarried	79	4.91 ± 1.60	2.82 ± 1.52	7.73 ± 2.5	**8.67 ± 3.47a**	9.18 ± 2.94	7.62 ± 2.79	25.47 ± 6.99	10.71 ± 2.57
	Married	251	4.55 ± 1.79	2.47 ± 1.49	7.02 ± 2.63	**10.05 ± 3.53b**	9.18 ± 3.09	7.54 ± 2.83	26.76 ± 6.44	10.69 ± 2.59
	Other	14	5.21 ± 1.37	1.93 ± 1.73	7.14 ± 2.25	**11.14 ± 3.134b**	9.5 ± 3.08	7.21 ± 3.77	27.86 ± 6.46	11.57 ± 2.59
	*p*-value^A^		0.126	0.061	0.1	**0.003**	0.927	0.886	0.230	0.458
Education level	Primary and below	72	**4.37 ± 1.70a**	**2.11 ± 1.43a**	**6.49 ± 2.46a**	**9.61 ± 3.446a**	**8.44 ± 2.88**	7.14 ± 2.77	**25.19 ± 5.03a**	**9.83 ± 1.96a**
	Junior high school	111	**4.4 ± 1.87ab**	**2.29 ± 1.42a**	**6.69 ± 2.68ab**	**9.86 ± 3.333a**	**9.2 ± 2.87**	7.85 ± 2.77	**26.9 ± 6.1a**	**10.78 ± 2.6ab**
	High school	94	**4.7 ± 1.74bc**	**2.87 ± 1.57b**	**7.64 ± 2.64ab**	**9.62 ± 3.744a**	**9.23 ± 3.07**	7.28 ± 2.93	**25.53 ± 7.08a**	**11.07 ± 2.64b**
	Junior college or above	67	**5.22 ± 1.42c**	**2.90 ± 1.50b**	**8.12 ± 2.15bc**	**10.88 ± 3.506b**	**9.91 ± 3.37**	7.87 ± 2.95	**28.66 ± 7.54b**	**11.1 ± 2.86b**
	*p*-value^C^		**0.008**	**0.001**	**0.001**	**0.011**	**0.044**	0.225	**0.01**	**0.008**
Chronic disease	No chronic disease	219	4.63 ± 1.73	2.58 ± 1.47	7.21 ± 2.6	9.8 ± 3.538	9.36 ± 2.89	7.7 ± 2.85	26.86 ± 6.55	10.67 ± 2.59
	With chronic disease	125	4.71 ± 1.77	2.44 ± 1.58	7.15 ± 2.6	9.74 ± 3.579	8.9 ± 3.3	7.27 ± 2.86	25.9 ± 6.61	10.82 ± 2.58
	*p*-value^B^		0.659	0.140	0.854	0.874	0.291	0.179	0.196	0.598
BMI	Underweight	50	4.60 ± 1.59	2.52 ± 1.61	7.12 ± 2.68	10.1 ± 3.524	8.6 ± 3.01	7.5 ± 2.53	26.2 ± 5.57	11.04 ± 2.67
	Healthy weight	171	4.70 ± 1.68	2.53 ± 1.42	7.22 ± 2.45	9.73 ± 3.558	9.11 ± 2.99	7.53 ± 2.97	26.36 ± 6.54	10.72 ± 2.56
	Overweight	78	4.82 ± 1.71	2.71 ± 1.59	7.53 ± 2.52	10.01 ± 3.5	9.51 ± 3.17	7.18 ± 2.74	26.71 ± 6.89	10.81 ± 2.53
	Obesity	45	4.29 ± 2.14	2.24 ± 1.60	6.53 ± 3.09	9.18 ± 3.657	9.6 ± 3.09	8.31 ± 2.92	27.09 ± 7.32	10.27 ± 2.68
	*p*-value^C^		0.622C	0.449	0.236	0.56	0.298	0.21	0.89	0.525

^A^Analysis of variance (ANOVA) was conducted, ^B^Mann–Whitney U test was conducted, ^C^Kruskal–Wallis test was conducted. Mean scores denoted by the same letter are not significantly different from each other at the 5% significance level based on Bonferroni *post hoc* analysis. Significant differences (*p* < 0.05) are in bold. PNK, procedural nutrition knowledge; DNK, declarative nutrition knowledge; IACC, information access capacity; IUC, information understanding capacity; IAPC, information application capacity; FHL, functional health literacy; DQI-I, Diet Quality Index-International.

For the health literacy status of rural residents, the overall FHL varies mainly by annual household income and education level. Regarding the three sub-dimensions of FHL, there are also differences in their influencing factors. Gender was identified as shaping the differences in residents’ IUC and IAPC. The education level contributes to the different IACC and IUC. Moreover, residents’ IUC and IACC are influenced by annual household income and marital status, respectively.

We identified significant differences in dietary variety between different education levels, that is, the higher the education level, the healthier the dietary behavior. The average DQI-I score for individuals in high school and above was over 11, while the average score for individuals in primary school and below was less than 10.

The results of the above analysis showed that male, elder, low-income, unmarried, and low-education groups performed significantly worse than their counterparts on one or more dimensions of knowledge, health literacy, and dietary behavior. Of these, male is showing significantly worse performance than female in three dimensions, namely nutrition knowledge, IUC, and IAPC. The 55 and older group performs significantly worse than the younger group in one dimension, i.e., DNK. The high-income group performs significantly better than the low-income group in five dimensions, i.e., PNK, DNK, nutrition knowledge, IUC, and FHL. The married group performs significantly better than the unmarried group in one dimension, i.e., IACC. Except for IAPC, the low-educated group was significantly worse than the high-educated group in the remaining dimensions. Given these findings, male, older adult, low-income, unmarried, and low-education groups are considered high-risk groups in terms of healthy diet and warrant prioritization in the design of intervention programs.

### 3.4 Relationship between nutrition knowledge and health literacy with dietary variety

Table 6 shows the results of the regression analysis. The VIF values were all below 1.5, indicating that there was no multicollinearity. The regression results showed that nutrition knowledge and health literacy could explain 31.5% of variance in dietary behavior. IACC, IUC, IAPC, and PNK significantly and positively affected DQI-I. Of these, the effects of IACC (*β* = 0.291) and IAPC (*β* = 0.277) on dietary behavior were the most influential. The coefficient of DNK on DQI-I was positive but not significant. After adding age, marital status, gender, annual household income, and education level as control variables, the effect of the above variables on DQI-I remains significant, showing that the test results are robust. The effects of control variables on dietary behavior were not significant.

**Table 6:** Multiple linear regression analysis results for dietary behavior.

	DQI-I			DQI-I		
Variables	*β*	*t*	VIF	*β*	*t*	VIF
Constant	0.029^**^	6.765		−0.055^**^	3.768	
IACC	0.291^**^	6.25	1.073	0.286^**^	5.968	1.128
IUC	0.211^**^	4.241	1.227	0.209^**^	4.116	1.263
IAPC	0.277^**^	5.778	1.134	0.272^**^	5.57	1.169
PNK	0.115^**^	2.45	1.087	0.107^**^	2.323	1.118
DNK	0.077	1.596	1.141	0.07	1.433	1.188
Age				0.05	0.97	1.279
Marital status				−0.005	−0.11	1.077
Gender				0.009	0.187	1.064
Annual household income				−0.039	−0.82	1.101
Education level				0.096	1.825	1.373
*R*^2^ = 0.315 *F*(5,338) = 31.146, *p* < 0.001				*R*^2^ = 0.323 *F*(10,343) = 15.87, *p* < 0.001		

^**^p < 0.05. PNK, procedural nutrition knowledge; DNK, declarative nutrition knowledge; IACC, information access capacity; IUC, information understanding capacity; IAPC, information application capacity; FHL, functional health literacy; DQI-I, Diet Quality Index-International.

## 4 Discussion

In this study, a validated questionnaire was developed to measure the nutrition knowledge (including PNK and DNK), FHL, and DQI-I of rural residents in northeastern China. Based on survey data through face-to-face approach, we were informed about the baseline status of dietary knowledge, health literacy and dietary behavior of rural residents. By examining the differences in different demographic characteristics, high-risk groups with poorer performance in terms of knowledge, literacy, and behavior were identified. In addition, factors associated with dietary behavior were also uncovered by evaluating the effect of dietary knowledge and health literacy. Based on these results, we were able to give evidence-based guidance on the priority content and populations for healthy dietary interventions.

### 4.1 The picture of consumers’ nutrition knowledge, FHL, and dietary behavior

The study showed the average correct scores of PNK, DNK and overall knowledge scores of rural residents in the three northeastern provinces were 66.57% (4.66/7), 42.17% (2.53/7) and 55.31% (7.19/13), respectively. This indicates that the level of nutritional knowledge of the residents is not sufficient and needs to be further promoted. This is consistent with the outcomes of other studies on Chinese residents, e.g., Hou et al. (56) and Zhang et al. (57). The relationship analysis between dietary knowledge and behavior showed that PNK had a positive effect on dietary behavior, while DNK had a non-significant effect on dietary behavior, indicating that different types of knowledge showed different effects on behavior and that the priority of different types of knowledge should be weighed in designing intervention programs.

The results of this study suggest that improvements in behavior may be insignificant if the intervention is targeted to residents’ descriptive knowledge. Previous studies that found a weak relationship between dietary knowledge and behavior may also have resulted from a failure to distinguish between different types of knowledge (58–60). Accordingly, integrating content related to procedural knowledge in intervention information development is an effective way to bridge the knowledge–behavior gap in dietary interventions.

The FHL score was generally low, and only 3% of the respondents showed excellent health literacy. This result is significantly lower than the health literacy of the residents of Denmark (35.2 ± 4.0, rang from 9 to 40) (61), Australia (Mean = 30.31, range from 9 to 40) (62) and Netherlands (Mean = 30.06, range from 9 to 40) (63). Li et al. (64) surveyed the Chinese population and found that the proportion of urban residents with adequate health literacy was 24%, while the proportion of the rural population with adequate health literacy in this study was only 20% (17% sufficient and 3% excellent). These suggest that the health literacy of Chinese rural residents is poor and needs to be improved. Furthermore, the ability to apply information to manage dietary was the lowest. FHL was evidenced to be significantly associated with the residents’ dietary behavior. Specifically, residents with stronger IACC, IUC, and IAPC had higher DQI-I scores. This is consistent with the findings of a systematic review, which showed that FHL was the most important predictor variable of dietary behavior (65). Some studies also proved that health literacy was a predictor of fruit and vegetable intake (66, 67). This signifies that diet-related health literacy is the priority to be addressed when designing intervention programs, including improving individuals’ ability to access, understand, and apply health information.

The dietary behavior met the recommendations (score = 15) accounted for only 15.41% of the residents, and nearly 50% of the individuals were those who had two food categories with less than the recommended intake. Among them, there was a high proportion (87.8%) of participants that met the recommended grain intake value. The reason for this phenomenon is that northeastern China is a major food-producing region where farmers mainly grow rice. The cost for farmers to obtain grain through self-sufficiency is relatively low. Besides, the proportion of fruits, dairy products and beans, and vegetables consumed up to the recommended values is quite low. Previous studies have also found that Chinese consumers’ intake of these food groups is grossly inadequate (68, 69). Fruits and vegetables can provide dietary fiber, which is strongly associated with a low incidence of cardiovascular disease and obesity (70). This implies that there is a great urgency to improve the dietary patterns of the Chinese residents, and intervention information should focus on increasing the population’s intake of specific foods (e.g., fruits, vegetables, beans, and dairy products).

### 4.2 Conditions under different demographic characteristics

This study revealed significant differences in dietary knowledge, health literacy, and behavior across demographic variables, which provides the empirical evidence for identifying high-risk groups in relation to healthy diets. One of the significant factors contributing to the differences in nutritional knowledge and FHL was gender. Females have more nutritional knowledge, information understanding and information application capacity than males. Dickson-Spillmann et al. (36) also found that PNK was significantly higher in females than in males. However, some previous studies found no significant relationship between health literacy by gender (71, 72) or that males have higher health literacy than females (73). The reason for this different outcome may be that the dietary decision makers in Chinese households are generally females, who spend more time and energy on food choices and therefore generate more health literacy than males (74). This also suggests that Chinese males are poor performers in terms of health behaviors and are a priority group for dietary interventions.

Age affects DNK, i.e., the elder the respondent, the lower the DNK, which is consistent with previous studies concluding that age is negatively associated with nutritional knowledge (36), but this differs from Hendrie, Coveney and Cox (75). These differences may be due to the fact that the questionnaire items are according to the latest healthy dietary recommendation guidelines, which differ from the past versions. Access to up-to-date knowledge can be a challenge for the elder community (76). Accordingly, this points to the elder population as a priority group for dietary interventions.

Previous studies have shown that residents with higher incomes have access to health information and services (67, 77, 78). Our study also found significant differences in nutritional knowledge and information understanding capacity between income levels, that is, those with higher incomes generally had more nutritional knowledge, including PNK and DNK, and higher health literacy. While lower income groups are the ones that need more attention in dietary interventions owing to poor performance in terms of knowledge and health literacy.

It has been shown that higher education levels not only help individuals to acquire knowledge and skills, but also enable them to better translate this information into health literacy (79, 80). Our findings also validated this finding and uncovered that those with higher education levels showed better in terms of dietary behavior, which is consistent with the Yang et al. (81). Furthermore, Kristal et al. (82) pointed to a relationship between the effectiveness of the dietary intervention and the years of education of the subjects. The educational level has been confirmed in several studies to have a significant positive effect on health behaviors (83, 84). In light of this study’s findings, individuals with lower education levels as a high-risk group deserve priority treatment in dietary intervention.

Marital status would affect the residents’ health literacy, mainly in the sense that married individuals have better ability to receive health information than unmarried ones. The reason for this may be that married individuals invest more energy in their family life, including paying attention to their family’s health, which leads these individuals to be more willing to obtain health information. This also means that unmarried residents need increased focus in dietary interventions.

### 4.3 Implications for designing dietary intervention

#### 4.3.1 Implications for designing intervention information

By investigating the nutrition knowledge, FHL, and dietary behavior of rural residents in northeastern China and analyzing the relationships between these factors, this study identifies priority intervention information and populations for health dietary promotion programs. We found that the nutrition knowledge, FHL, and dietary behavior is not well developed and needs to be improved. Among them, PNK and FHL have significant positive effects on dietary variety. In order to more effectively promote healthy dietary behaviors, the intervention information needs to involve knowledge of how to more efficiently and rationally implement healthy dietary and improve individual health literacy. Dietary intervention programs in China now mostly introduce residents to what constitutes a healthy diet, i.e., DNK, which helps improve DNK but has limited effect on behavior improvement. Developing Internet and mobile phone app–based dietary guidelines is also essential to decrease the difficulty of accessing, understanding, and applying information.

#### 4.3.2 Implications for targeting high-risk population groups

Moreover, by understanding the performance of individuals under different demographic characteristics it is also possible to identify priority populations for intervention. Groups that are male, older adult, low-income, unmarried, and low education levels should be given more attention because they perform worse in terms of knowledge, health literacy, and dietary behaviors. The number of mobile Internet users in China is nearly one billion (85), which allows the use of big data technology to push healthy diet information to priority groups.

### 4.4 Limitations and future studies

Although we have made an in-depth consideration of the research design there are still some limitations in this study. First, we selected only DQI-I as a marker for dietary behavior assessment. Other markers could be added for dietary behavior assessment in future studies, such as the proportion of macro-energy supply and micronutrient intake. Second, this study focused only on nutritional knowledge and FHL as influencing factors of dietary behavior. However, in previous studies it has also been revealed that there are other factors that may have influenced dietary behaviors, such as social network type, self-efficacy, and food availability (86, 87). In coming studies, psychosocial models and environmental factors could be considered to evaluate dietary behavior. Additionally, this study used the FFQ as an instrument to investigate dietary behavior. Although this measurement method is low-cost and valid, its accuracy is slightly lacking. More accurate survey tools, such as logs or real-time images, can be chosen to record dietary behaviors. Finally, we only surveyed the northeastern region of China, and although the northeastern region is quite representative, a national survey could be considered in the future to get a clearer picture of the dietary situation of Chinese residents.

## 5 Conclusion

This study showed that the dietary behavior, nutrition knowledge, and health literacy of rural residents in China need to be improved. FHL is an important factor affecting dietary behavior, and more attention should be paid to this issue in dietary behavior interventions. As a different type of nutrition knowledge, procedural nutrition knowledge showed a significant positive influence on dietary behavior, whereas declarative nutrition knowledge was not significantly influenced with dietary behavior. The promotion of procedural nutrition knowledge should be reinforced in the promotion of healthy diets going forward. The results of differences analysis demonstrated that demographic variables affect individual nutritional knowledge, health literacy, and dietary behavior and identified males, older adult, unmarried, low-income, and low-education populations as high-risk group. This provides evidence-based guidance for prioritizing information and populations for healthy dietary interventions.

## Data availability statement

The raw data supporting the conclusions of this article will be made available by the authors, without undue reservation.

## Ethics statement

The studies involving humans were approved by School of Biological and Agricultural Engineering, Jilin University. The studies were conducted in accordance with the local legislation and institutional requirements. The participants provided their written informed consent to participate in this study.

## Author contributions

LB: Conceptualization, Data curation, Formal analysis, Methodology, Validation, Writing – review & editing. HT: Resources, Formal analysis, Investigation, Writing – original draft. MW: Supervision, Conceptualization, Writing – review & editing, Funding acquisition. All authors contributed to the article and approved the submitted version.

## Footnotes

^1^On November 1, 2022, 1 U.S. dollar equals approximately 7.264 RMB.
