# Supplementary material for: Dietary behaviors of rural residents in northeastern China: implications for designing intervention information and targeting high-risk population
Source: Front Public Health. 2024 Jan 23;12:1239449. doi: 10.3389/fpubh.2024.1239449 (PMC10883159; doi:10.3389/fpubh.2024.1239449)
Supplement: Supplementary file 1 [file Data_Sheet_1.docx]

Fig S1 The schematic diagram of standard food weight


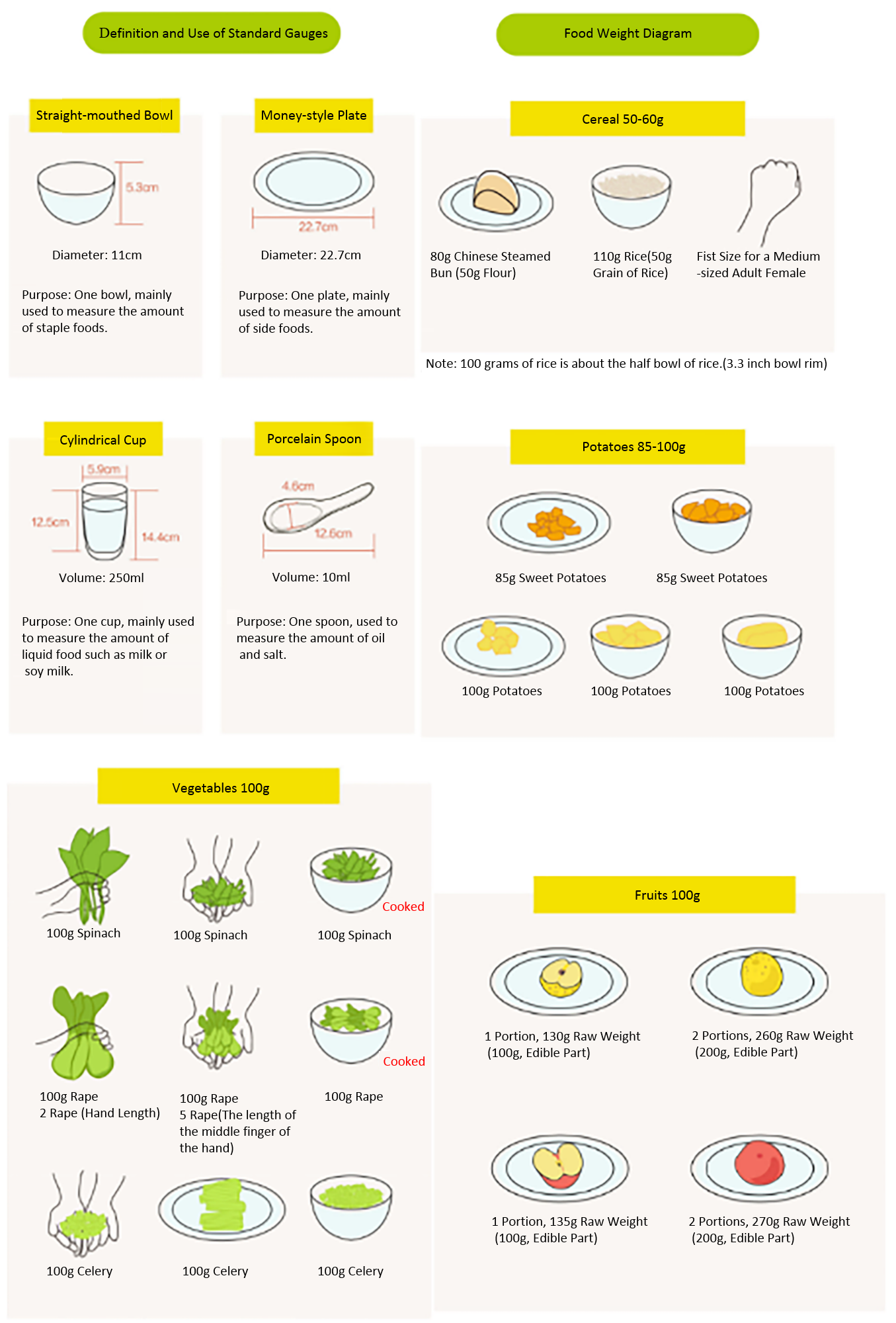


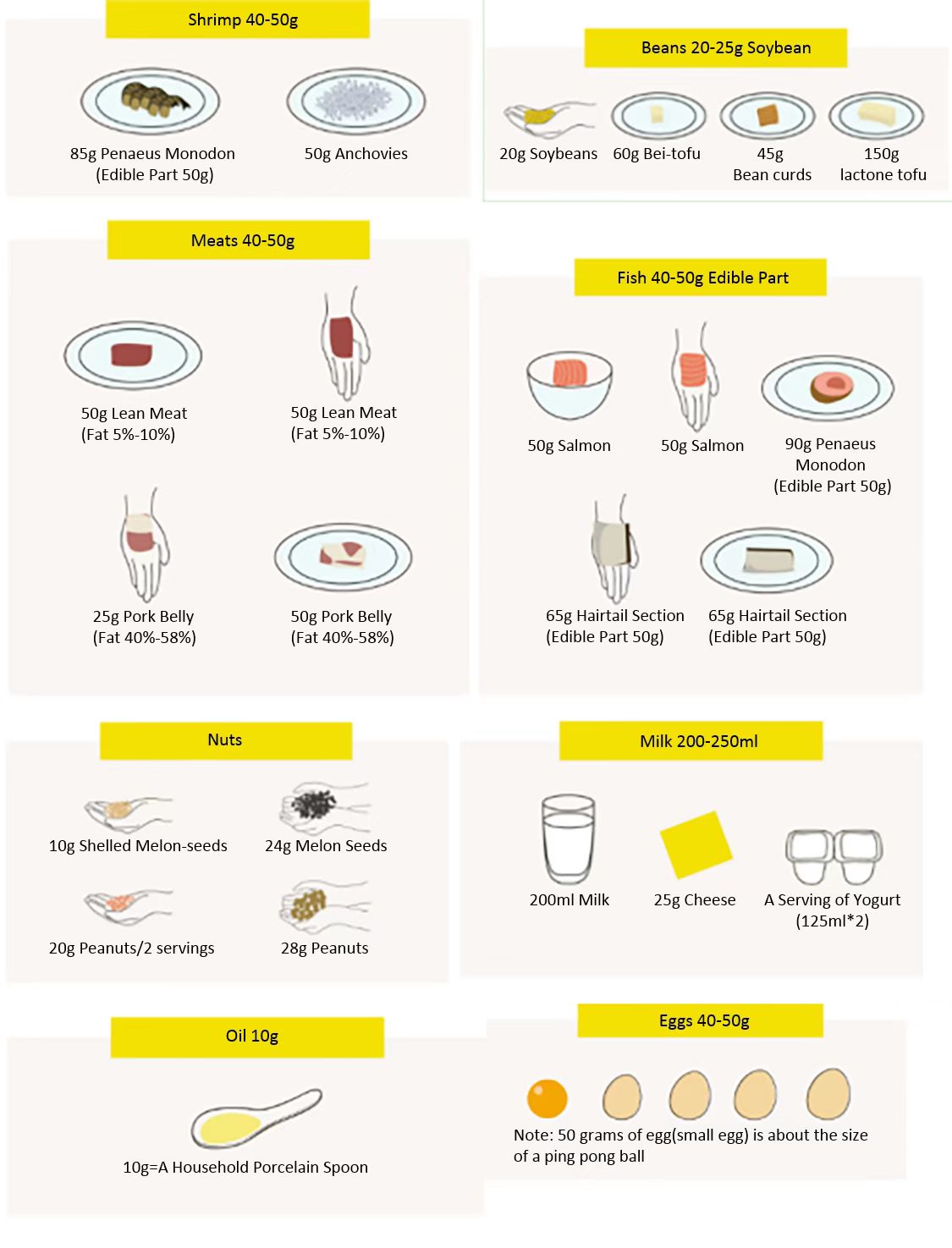


Table S1 Dietary intake questionnaire

| **Category（Y）** | **NO.** | **Name** | **Frequency** | | **Average dose per session （M）** |
| --- | --- | --- | --- | --- | --- |
|  |  |  | **Time/day（X）** | **Time/week(W）** |  |
| Meat/Poultry/Fish/Eggs | 1 | Pork |  |  |  |
|  | 2 | Beef |  |  |  |
|  | 3 | Mutton |  |  |  |
|  | 4 | Chicken |  |  |  |
|  | 5 | Duck |  |  |  |
|  | 6 | Egg |  |  |  |
|  | 7 | Fish |  |  |  |
|  | 8 | Shrimp |  |  |  |
| Beans/Dairy products | 9 | Soybean milk |  |  |  |
|  | 10 | Doufu |  |  |  |
|  | 11 | Dried beancurd |  |  |  |
|  | 12 | Milk |  |  |  |
|  | 13 | Beans/Dairy products |  |  |  |
| Grains | 14 | Rice |  |  |  |
|  | 15 | Noodles |  |  |  |
|  | 16 | Mantou |  |  |  |
|  | 17 | Corn |  |  |  |
|  | 18 | Other grains |  |  |  |
| Fruits | 19 | Apple |  |  |  |
|  | 20 | Banana |  |  |  |
|  | 21 | Watermelon |  |  |  |
|  | 22 | Peach |  |  |  |
|  | 23 | Other fruits |  |  |  |
|  | 24 | Other fruits |  |  |  |
| Vegetables | 25 | Chinese cabbage |  |  |  |
|  | 26 | Cauliflower |  |  |  |
|  | 27 | Eggplant |  |  |  |
|  | 28 | Tomato |  |  |  |
|  | 29 | 0ther vegetables |  |  |  |
|  | 30 | other vegetables |  |  |  |
| Others | 31 | Others |  |  |  |
|  | 32 | Others |  |  |  |
